# The Tumor Microenvironment of Pediatric Sarcoma: Mesenchymal Mechanisms Regulating Cell Migration and Metastasis

**DOI:** 10.1007/s11912-019-0839-6

**Published:** 2019-08-15

**Authors:** Monika Ehnman, Wiem Chaabane, Felix Haglund, Panagiotis Tsagkozis

**Affiliations:** 1 Department of Oncology-Pathology, Karolinska Institutet, Stockholm, Sweden; 2 PO Bröst- endokrina tumörer och sarkom, Tema Cancer, BioClinicum J6:20, Karolinska University Hospital, Visionsgatan 4, 171 64 Solna, Sweden; 3 Section of Orthopaedics, Department of Molecular Medicine and Surgery, Karolinska Institutet and Department of Orthopaedics, Karolinska University Hospital, Stockholm, Sweden

**Keywords:** Pediatric sarcoma, Osteosarcoma, Rhabdomyosarcoma, Ewing sarcoma, Tumor microenvironment, Metastasis, EMT, MET, Extracellular matrix, Stroma, TGFβ, PDGF, CXCR4

## Abstract

**Purpose of Review:**

This review presents a selection of regulatory molecules of tumor microenvironmental properties and metastasis. Signaling pathways controlling mesenchymal biology in bone and soft-tissue sarcomas found in children and adolescents are prioritized.

**Recent Findings:**

The tumor microenvironment of pediatric tumors is still relatively unexplored. Highlighted findings are mainly on deregulated genes associated with cell adhesion, migration, and tumor cell dissemination. How these processes are involved in a mesenchymal phenotype and metastasis is further discussed in relation to the epithelial to mesenchymal transition (EMT) in epithelial tumors. Cell plasticity is emerging as a concept with impact on tumor behavior.

**Summary:**

Sarcomas belong to a heterogeneous group of tumors where local recurrence and tumor spread pose major challenges despite intense multimodal treatments. Molecular pathways involved in the metastatic process are currently being characterized, and tumor-regulatory properties of structural components, and infiltrating, non-malignant cell types should be further investigated.

## Introduction

Pediatric tumors typically present with an overall low mutational burden but often with recurrent chromosomal aberrations [1••]. Detection of identified pathognomonic fusion genes is for relevant sarcoma subtypes already used in the diagnostic routine, and there is a link between fusion gene status and worse outcome among non-metastatic patients [2]. Clearly, genetic and epigenetic changes in tumor cells are of importance for tumor progression and these changes also contribute to modifications of the tumor microenvironment.

Numerous studies have by now elegantly illustrated how activated non-malignant stromal cells control matrix stiffness; become contractile and pro-invasive [3, 4]; and importantly, how these activities affect drug efficacy and metastasis [5]. However, the majority of studies on the tumor microenvironment have been carried out in epithelial entities of adulthood, such as breast carcinomas. Mesenchymal tumors in children are fundamentally different, and the function of non-malignant cells in these tumors is less characterized. In untreated tumors, activated stromal cells are less likely to form distinct compartments as they often do in epithelial tumors. Instead, they intermix with tumor cells, immune cells, and other cell types in a tumor-surrounding pseudocapsule, or possibly support endothelial tube formation during angiogenesis. Whether their structural support is required during sarcoma progression has not been systematically investigated, and the absence of specific markers for non-malignant stromal cells in mesenchymal tumors makes such studies more challenging.

How metastatic spread of pediatric tumors is controlled by the tumor microenvironment is still an early field of research. Common sarcoma subtypes of childhood include osteosarcoma, Ewing sarcoma, and rhabdomyosarcoma. The prognosis of these tumors is relatively favorable compared to many sarcomas in adults, but metastatic spread is still problematic. Osteosarcoma principally originates from bone and is likely the subtype where the tumor microenvironment has been most characterized. Ewing sarcoma arises in either bone or soft tissue, whereas rhabdomyosarcoma is a soft-tissue sarcoma displaying signs of a myogenic program with skeletal muscle features. Recent findings from mainly these three pediatric sarcomas provide the basis for this review on the tumor microenvironment and mesenchymal mechanisms regulating cell migration and metastasis.

## Clinical Features and Treatment Principles

### Symptoms

Bone sarcomas are generally characterized by pain, whereas a painless mass is more common in soft-tissue sarcomas. A palpable mass may or may not be present in bone tumors. Constitutional symptoms such as malaise, wasting, or generalized signs of inflammation with fever are only occasionally observed if the tumors are large and necrotic [6, 7]. In some cases, the disease is spread at diagnosis with obvious metastases.

### Pre-Treatment Staging

Rhabdomyosarcomas and Ewing sarcomas are high-grade tumors, whereas osteosarcomas can be of high grade or low grade. Staging is based on the TNM or the musculoskeletal tumor society system, and the clinical group system is applicable in rhabdomyosarcoma [8, 9]. About 75–90% of pediatric sarcomas initiate as localized disease, but micrometastases are assumed to be present in practically all cases. This is demonstrated in historical survival data of patients who did not receive chemotherapy, where radical surgery was associated with good local control of the disease, but low overall survival [7, 10].

The three major types of pediatric sarcomas have a similar pattern of metastasis, with hematogenous spread as the classic route of dissemination. Lungs are the most prominent metastatic site, thereafter bone and bone marrow. Less common sites are lymph nodes (especially for rhabdomyosarcomas), viscera, and soft tissues [8, 11]. The fact that micrometastatic disease is a rule rather than an exception in high-grade pediatric sarcomas suggests that the underlying mechanisms of tumor cell dissemination are active in early stages of the disease.

### Treatment

Modern treatment regimens include early systemic chemotherapy to eradicate micrometastatic disease. This is combined with local excision of the primary tumor and macrometastases when feasible. Most patients are given neoadjuvant chemotherapy for local disease control and continue with additional cycles of chemotherapy after surgery [9, 12, 13]. Radiotherapy is given particularly when surgical margins are poor, and for local control of radiosensitive tumors, such as Ewing sarcomas and rhabdomyosarcomas, when the primary tumor is inoperable.

## Genetics, Molecular Diagnostics, and Prognostic Factors

### Fusion Gene Status

Detection of tumor-specific translocations is often diagnostically useful in pediatric sarcomas [14]. *EWS-ETS* gene fusion variants are found in Ewing sarcomas, and similarly, the most common fusion genes associated with alveolar rhabdomyosarcoma are *PAX3-FOXO1* and *PAX7-FOXO1*. Accordingly, the two major subtypes of rhabdomyosarcoma, with the alveolar subtype being more aggressive, are with some exceptions distinguishable with modern techniques. However, even though embryonal rhabdomyosarcomas typically develop earlier along the developmental program compared to the alveolar subtype, they remain clinically and molecularly indistinguishable from fusion gene-negative alveolar rhabdomyosarcomas [15••, 16].

### Clinicopathological Factors Associated with Prognosis

The prognosis of pediatric sarcoma is dependent on a series of factors, including size and site of the primary tumor and age of the patient [9, 17, 18]. In this context, the initial disease burden is crucial, where children presenting with localized disease have a much better prognosis than children with evident tumor spread. The single most important treatment-associated factor for patient outcome is response to chemotherapy. In bone sarcomas, this is routinely measured as the degree of necrosis after neoadjuvant chemotherapy [18, 19]. Several histopathological protocols exist [20–23]. Poor responders have inferior oncologic outcome and are defined, according to the most widely accepted criteria, as those with less than 90% chemotherapy-induced tumor necrosis. Another treatment-associated factor of importance is the quality of surgical margins [24].

## Cell Migration and Metastatic Dissemination

When tumor cells initiate the multistep process of metastasis, they have begun a journey where adaptation to foreign tissue microenvironments is essential for survival. There are still many unknowns about selection processes during disease progression, where only some sarcoma cells reach anatomically distant organs and successfully metastasize. The discussion below is focused on mesenchymal cell plasticity and cell adhesion molecules involved in cell migration and metastasis. Notably, mesenchymal properties in sarcoma are regulated by multiple developmental signaling pathways, and some of these were recently reviewed elsewhere [25].

### Migration and Cell Adhesion

Cell migration can be broadly categorized as collective cell migration (epithelial cancers) or individual cell migration (sarcoma). The mesenchymal cell migration of sarcomas can involve both single cells or cells in chains and is typically regulated by the extracellular matrix (ECM), various integrins, and proteases. Cadherins, which form adherens junctions, are broadly implicated in direct cell-cell contacts in multicellular organisms. Mesenchymal adherens junctions are expected to be more transient compared to the epithelial counterpart, and the stability is partly controlled by endocytosis and regulation of the cytoskeleton.

It is well known that downregulation of E-cadherin is essential during the cellular program of epithelial to mesenchymal transition (EMT), and its upregulation is conversely linked to the mesenchymal to epithelial transition (MET) during establishment of distant metastasis. A mesenchymal to amoeboid transition (MAT) has also been described in osteosarcoma during transendothelial migration [26•]. Recently, when mesenchymal traits were reviewed in epithelial cancers, a partial EMT was concluded to be beneficial for the tumor-initiating ability, whereas drug resistance plateaued, and was maintained, with further activation of the EMT program [27]. Invasiveness was most effective when strong activation of EMT led to single cell migration instead of the classic multicellular carcinoma cell migration.

The process of EMT in sarcoma is by definition less obvious, but E-cadherin expression is also here known to reduce anchorage-independent growth and spheroid formation [28•]. The tight junction protein claudin-1 is another example of an epithelial differentiation marker found in sarcoma [29]. Evidently, epithelial markers in sarcomas have been shown to correlate with improved patient outcome [30, 31, 32•]. What may sound contra-intuitive, though, is that forced expression of mesenchymal-associated adhesion molecules has been shown to inhibit cell migration and metastasis in osteosarcoma while being associated with bone metastasis in carcinomas and Ewing sarcoma [33, 34]. However, both cadherin-11 and N-cadherin are highly expressed in normal osteoblasts, where they regulate cell function and differentiation. Therefore, a subtype-specific tumor microenvironment may explain why reduced levels are suggested to be of importance during disease progression and metastasis in osteosarcoma [35].

Cadherin switching and induced expression of the EMT marker N-cadherin is associated with morphological changes toward a mesenchymal phenotype with migratory and invasive properties in malignant cells of epithelial origin. Similar mechanisms have also been reported in mesenchymal malignancies. For example, induction of N-cadherin and alpha9-integrin increases cellular invasion in a Notch-dependent manner in rhabdomyosarcoma [36]. Notch signaling is a developmental pathway generally known to participate in sarcoma progression at multiple levels with regulatory functions on cell migration, stemness, and angiogenesis. In osteosarcoma, endothelial cells and pericytes have been suggested as sources for Notch activation [37]. Importantly, deregulated developmental processes are believed to play a major role in pediatric sarcomas.

By now, there are numerous publications on disease-regulatory roles of epithelial and mesenchymal markers involved in cell movements in pediatric sarcoma. Preussner et al. recently explored the importance of epithelial/mesenchymal states in the context of tumor cell plasticity in a genetic mouse model of rhabdomyosarcoma [38••]. In a genome-unstable, tumor-prone microenvironment of regenerating muscle, muscle stem cells initiated successful tumorigenesis by a MET-like process via zygotic Dux transcription factors. In the experimental setting, overexpression of Duxbl in wild-type muscle stem cells resulted in cadherin expression, immortalization, and an ability to form tumors. The authors further linked Dux transcription factors to stem cell expression profiles in tumors of germ cell or epithelial origin. Hereby, this study provides additional evidence for tumor heterogeneity and detection of stem cell traits in rhabdomyosarcoma as indicated in earlier literature [39, 40].

### Models of Dissemination

Invasive properties of primary tumors may not always imply a capacity to establish distant metastases followed by decreased overall survival. A recent EpSSG study demonstrated that small pulmonary nodules at diagnosis could be present in over 20% of otherwise localized rhabdomyosarcoma, but these nodules were not found to impact survival [41]. Clearly, what determines metastatic outgrowth before, during, or after local or systemic therapy is essential to understand at multiple levels.

The traditional linear progression model of metastasis is based on the assumption that genetic mutations accumulate in the tumor over time, and eventually, subclonal populations acquire metastatic features. Compelling evidence instead favors an early dissemination process, with more effective colonization, and pathways of parallel progression [42]. This latter model is well in line with that the mutational landscape at metastatic sites can be fundamentally different from the primary tumor.

Regardless of whether dissemination occurs early or late during tumor progression, it also involves different anatomic locations for metastatic outgrowth in a tumor type-specific manner. The organotropic model of metastasis describes how organ tropism or pre-metastatic niches facilitate successful metastasis according to a modified notion of the classic seed and soil hypothesis, where certain tumor cells (seed) have an affinity for the milieu (soil) of certain organs [43]. In contrast, the anatomical/mechanical model considers a filter and flow principle of metastatic clones, where anatomical barriers control the seeding [44]. The relative contribution of each model in various tumor types can be debated, but it is now clear that circulating tumor cells is a frequent phenomenon and metastasis is generally considered to be an inefficient process [44].

## Regulators of Metastasis in the Mesenchymal Tumor Microenvironment

**Fig. 1:**
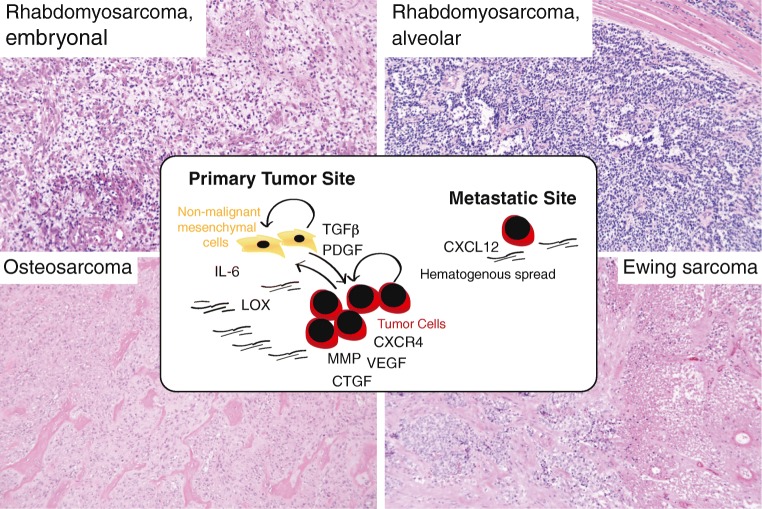
Schematic summary and representative microphotographs of the histomorphological presentation of childhood sarcomas. In front: key molecular pathways discussed in the text. Top left: embryonal rhabdomyosarcomas (ERMS) generally present as primitive small blue round mesenchymal cells with varying degrees of myogenic differentiation (commonly identified with routine immunohistochemical markers). Tumors associated with an epithelial mucosa are commonly referred to as botryoid ERMS and have a generally better prognosis. RMS may also present as anaplastic/pleomorphic (TP53 mutation associated) or sclerotic/spindle cell (MYOD1 mutation associated) variants with poorer prognosis. Top right: alveolar rhabdomyosarcomas (ARMS) are small blue round cell tumors, with nests or sheets of tumor cells growing in alveolar spaces. Solid cases lack alveolar patterns and only present with fibrovascular septa. Bottom left: osteosarcomas (OS) are diagnosed based on clinical, radiological, and histopathological features. The tumor cells produce pink osteoid matrix (immature neoplastic bone) and permeate adjacent cortical bone or soft tissue. In the classic high-grade intramedullary OS, the tumor cells are pleomorphic and hyperchromatic and may be dominated by osteoblastic (represented in the figure), chondroblastic, or fibroblastic features. Bottom right: Ewing sarcomas (ES) are small blue undifferentiated round cell tumors that usually have cytoplasmic glycogen vacuoles and rarely a stromal component. The figure depicts a neoadjuvant-treated tumor with partial necrosis (defined as a poor responder according to the study protocol criteria)

The sarcoma tumor microenvironment can vary extensively with subtype, anatomic location, age, gender, genomic complexity, and prior treatment. Molecules of relevance from a mesenchymal stroma perspective are reviewed below with a particular focus on those regulating cellular transition states and migration in pediatric sarcoma (schematic summary in Fig. 1). The importance of vascular cells, immune cells, and immunotherapy in sarcoma has recently been reviewed elsewhere [45–47].

### Key Players in the Sarcoma Tumor Microenvironment

The tumor microenvironment is broadly composed of malignant cells and non-malignant stromal cells, vascular cells, and immune cells. Structural ECM proteins (such as collagens) and matricellular proteins (such as osteopontin) provide support and signaling cues of importance for cell movements. Several ECM-associated proteins are regulated by transforming growth factor beta (TGFβ) and act as potential biomarkers [48]. A unique feature of sarcomas is that the distinction between malignant cells and stromal cells is particularly vague due to the mesenchymal origin of the tumor cells. Cellular transdifferentiation of bone marrow-derived mesenchymal stem cells can also occur, which is particularly characterized as important in osteosarcoma progression [26•].

A hypoxic tumor microenvironment generally contributes to tumor progression, and the impact of intratumoral oxygen gradients has been studied in sarcoma cell invasion [49]. Hypoxia-induced HIF1α activates the SDF1-CXCR4 signaling axis and the observed elevated levels of the chemokine receptor CXCR4 persist when cells return to normoxic conditions [50•]. In sarcoma, there is evidence for that the SDF-1 ligand induces chemotaxis across membranes, adhesion to endothelial cells, and matrix metalloproteinase 2 (MMP-2) expression [51]. Both MMP-2 and MMP-9 have been suggested as prognostic markers and associate with metastasis in osteosarcoma [52, 53].

CXCR4 expression is detected in 67% of osteosarcomas and correlates with vascular endothelial growth factor (VEGF) expression and decreased patient survival [54, 55]. A correlation with decreased patient survival is also reported in rhabdomyosarcoma [56]. By now, there are numerous tumor settings where CXCR4-positive malignant cells are mechanistically likely to metastasize to SDF1 (CXCL12)-expressing organs, such as the bone marrow [51]. The unique bone marrow microenvironment, with resident stem cells and progenitor cells, in turn has a propensity to attract and support disseminating tumor cells of different origin. Studies further demonstrate that local interaction with, or recruitment of, bone marrow-derived mesenchymal stem cells promotes primary tumor growth and invasion [57]. One proposed mechanism of action of mesenchymal stem cells in the tumor microenvironment is to contribute to stemness and chemoresistance via the NFκB pathway and IL6 secretion [58].

### Extracellular Matrix and Associated Proteins

Convincing work from Weaver and others have with time contributed to an increased basic understanding about how matrix stiffness and physical environments around cancerous cells matter for tumor progression [59]. In sarcomas, molecular findings demonstrate that the mechanical and chemical properties of the tumor microenvironment act together in a feedback loop to accelerate sarcoma motility and metastasis [60]. However, ECM proteins often have pleiotropic effects in the tumor microenvironment and must be considered in a context-specific manner when it comes to organ and tissue type.

This can be exemplified by lysyl oxidases (LOX), which are considered to be powerful regulators of structural modifications in normal connective tissue, fibrotic disease, and cancer. The LOX family consists of catalyzing enzymes involved in cross-linking of collagen and elastin in the tumor microenvironment. By now, numerous studies have demonstrated an active role of LOX family members in tumor progression and metastasis in tumor types of different origin. There are also reports on tumor-suppressive activities, for example in osteosarcoma [61]. In Ewing sarcoma, the *EWS-FLI* oncoprotein downregulates LOX and the reported tumor-suppressive activities have been linked to a propeptide domain [62]. Both LOX and LOXL1 contain prodomains and are processed extracellularly in contrast to the rest of the family members LOXL2, LOXL3 and LOXL4. For the mature protein to become active, proteolytic removal of its N-terminal LOX-propeptide, LOX-PP, is required.

Thrombospondin-1 (TSP1) represents a classic example of a glycoprotein in the tumor microenvironment and is commonly recognized for its anti-angiogenic functions and impact on tumor cell invasion via multiple cell surface molecules and matrix metalloproteinase interactions [63, 64]. Its pro-adhesive activities in osteosarcoma have been linked to the α4β1 integrin [65]. Since trabectedin was approved for treatment of advanced or metastatic soft-tissue sarcoma, several drug mechanisms of actions have been proposed, among others, anti-angiogenic activities on endothelial cells and upregulation of TSP1 [66]. The same study illustrated impaired ECM remodeling due to an increased tumor microenvironmental synthesis of tissue inhibitor of metalloproteinases 1 and 2. To what extent TSP1 can act as a regulator of angiogenesis-dependent dormancy remains to be seen.

### Mesenchymal Growth Factors

Cell proliferation and differentiation are often tightly linked processes and regulated by growth factors such as TGFβ, platelet-derived growth factor (PDGF), and fibroblast growth factor (FGF) in mesenchymal stem cells [67]. TGFβ is particularly known as a key regulator of the EMT phenomenon and the associated tumor progression in multiple tumor types. In osteosarcoma, high levels of TGFβ correlate with grade, chemoresistance, and presence of metastases [68, 69]. Similarly, overexpression of the downstream EMT transcription factors, such as Snails, ZEBs, or Twist, promotes tumor cell spread [70], while overexpression of the inhibitory transcription factor Smad7, or pharmacological inhibition, prevents disease progression [71–74]. However, genetic manipulations and in vivo analyses also demonstrate potential tumor-suppressive roles of TGFβ signaling in sarcoma [75].

Members of the TGFβ family act on different cell types in the tumor microenvironment. The TGFβ co-receptor endoglin is for example considered to be a vessel marker in tumor biology but is also expressed by malignant cells and has been linked to tumor cell plasticity and worse patient survival in Ewing sarcoma [76]. Other TGFβ-regulated factors in angiogenesis include VEGF and connective tissue growth factor (CTGF) [77]. VEGF expression has been associated with vessel density and decreased disease-free survival of osteosarcoma patients [78, 79]. CTGF was recently shown to promote angiogenesis, increase MMP-2/3 expression and cell migration in osteosarcoma, whereas knockdown of CTGF reduced lung metastasis in an experimental mouse model [80–82]. Other studies have shown that CTGF can increase drug resistance in osteosarcoma as well as regulate VEGF production from fibroblasts [83, 84].

The TGFβ pathway also participates in the selective suppression of the immune system [85, 86]. Osteosarcoma cells are able to control the recruitment and differentiation of infiltrating immune cells and establish a local immune tolerant microenvironment, allowing tumor progression [87]. Experiments have further shown that the immune response in osteosarcoma can be restored by combining an anti-TGFβ antibody with dendritic cells [88]. A novel mechanism by which tumors escape surveillance by the innate immune system was recently described by Gao et al. using a model system of methylcholanthrene (MCA)-induced fibrosarcoma. The study suggested that TGFβ-driven tumor immunoevasion included conversion of anti-tumoral NK cells into type 1 innate lymphoid cells with lost ability to control local tumor growth and metastasis [89].

Another developmental signaling pathway that is potentially activated during sarcomagenesis is the PDGF pathway. Recently, PDGF signaling was shown to play a role in maintaining cancer stem cell phenotypes such as self-renewal, invasion, and chemotherapy resistance in sarcoma [40, 90•]. Higher levels of phosphorylated PDGFRα/β and EMT proteins were detected in spheroid cultures (enriched for cancer stem cells), while the PDGFRα/β-targeting tyrosine kinase inhibitor imatinib reduced migration and invasion up to 80% and reduced expression of EMT proteins. These findings are in line with some of the previously reported oncogenic mechanisms of action of PDGF signaling, including autocrine stimulation of tumor cells, paracrine stimulation of stromal cells, promotion of angiogenesis, and regulation of the tumor interstitial fluid pressure (IFP), which controls the influx and eflux of agents [91–93].

In general, members of the PDGF family can be linked to primary tumor growth, metastasis, drug resistance, and poor clinical outcome in malignances of different cellular origin, but the role of PDGF activity in different sarcoma subtypes remains unclear [94]. Genetic aberrations of PDGF receptors are only detected in about 2% of pediatric cancers [1••]. Still, PDGF ligands and/or receptors are frequently expressed in rhabdomyosarcoma, osteosarcoma, and Ewing sarcoma and correlate with clinical outcome [40, 95–99]. Interestingly, *PAX3-FOXO1* (alveolar rhabdomyosarcoma), and *EWS-ETS* (Ewing sarcoma) are both examples of fusion genes with capacity to experimentally induce expression of PDGF family members [95, 100].

Notably, identified resistance mechanisms to therapeutic agents in sarcoma have included deregulated PDGF signaling. A recent example of this is the reported feedback interaction between CXCR4 and PDGF signaling in Ewing sarcoma, where high expression of CXCR4 correlates with metastasis and poor patient survival [101, 102•]. When tumor cells were treated with a CXCR4-targeting agent, compensatory activation of PDGFRβ led to increased proliferation that was counteracted by multi-kinase inhibitor treatment with dasatinib. Another report, from rhabdomyosarcoma, has identified amplified, overexpressed, and constitutively activated PDGFRα as an acquired resistance mechanism to an agent targeting insulin-like growth factor I receptor (IGF-IR) [103]. Altogether, these reports highlight the need for investigating mechanisms of action of anti-cancer agents to identify suitable combination treatments.

## Challenges and Future Directions

Oncologic treatment of pediatric sarcomas has classically relied on chemotherapy. Various agents have been used, and these have in common the preferential cytotoxicity against the malignant cells of the primary tumor and any metastatic site. We are now entering a new era in oncology, mainly characterized by the introduction of combination treatments and targeted therapy directed against malignant cells and/or cells of the tumor microenvironment. Such examples include imatinib for dermatofibrosarcoma protuberans and gastrointestinal stromal tumors and pazopanib for metastatic non-adipocytic soft-tissue sarcoma [94].

Other novel sarcoma treatments include trabectedin, which has been approved by the European Medicines Agency (EMA) for treatment of soft-tissue sarcomas in adults. Apart from direct activity on malignant cells, trabectedin modulates the phenotype of tumor-associated macrophages. Another EMA-approved treatment regimen targeting macrophages is muramyl tripeptide (mifamurtide), which is used for treatment of osteosarcoma. Immunotherapy has recently emerged as a promising strategy to modulate immune cell activity in subsets of patients but is still under early investigation in sarcoma. Therapeutic modification of the immune response in the pediatric sarcoma microenvironment is also explored using tumor vaccines. How effective such treatments would be remains to be determined.

## Conclusion

An increased understanding about tumor microenvironmental activities in pediatric sarcoma progression is essential for improving patient outcome and quality of life. Studies in common cancers of epithelial origin have been useful in the identification of candidate molecular pathways involved in metastasis and therapeutic resistance in sarcoma. However, the mesenchymal origin of sarcomas makes them unique and cellular processes like EMT and MET cannot be discussed in the same manner as in epithelial tumors. The heterogeneity between, and within, sarcoma subtypes is also particularly challenging. Consequently, how findings from other settings can be translated to pediatric sarcoma remains to be further explored.
